# Children with Moderate Acute Malnutrition with No Access to Supplementary Feeding Programmes Experience High Rates of Deterioration and No Improvement: Results from a Prospective Cohort Study in Rural Ethiopia

**DOI:** 10.1371/journal.pone.0153530

**Published:** 2016-04-21

**Authors:** Philip James, Kate Sadler, Mekitie Wondafrash, Alemayehu Argaw, Hanqi Luo, Benti Geleta, Kiya Kedir, Yilak Getnet, Tefera Belachew, Paluku Bahwere

**Affiliations:** 1 Valid International, Oxford, United Kingdom; 2 Population and Family Health Department, College of Public Health and Medical Sciences, Jimma University, Jimma, Ethiopia; 3 Department of Food Safety and Food Quality, Faculty of Bioscience Engineering, Ghent University, Ghent, Belgium; 4 Save the Children International, Addis Ababa, Ethiopia; 5 Research Center in Epidemiology, Biostatistics and Clinical Research, School of Public Health, Free University of Brussels, Brussels, Belgium; University of Lethbridge, CANADA

## Abstract

**Background:**

Children with moderate acute malnutrition (MAM) have an increased risk of mortality, infections and impaired physical and cognitive development compared to well-nourished children. In parts of Ethiopia not considered chronically food insecure there are no supplementary feeding programmes (SFPs) for treating MAM. The short-term outcomes of children who have MAM in such areas are not currently described, and there remains an urgent need for evidence-based policy recommendations.

**Methods:**

We defined MAM as mid-upper arm circumference (MUAC) of ≥11.0cm and <12.5cm with no bilateral pitting oedema to include Ethiopian government and World Health Organisation cut-offs. We prospectively surveyed 884 children aged 6–59 months living with MAM in a rural area of Ethiopia not eligible for a supplementary feeding programme. Weekly home visits were made for seven months (28 weeks), covering the end of peak malnutrition through to the post-harvest period (the most food secure window), collecting anthropometric, socio-demographic and food security data.

**Results:**

By the end of the study follow up, 32.5% (287/884) remained with MAM, 9.3% (82/884) experienced at least one episode of SAM (MUAC <11cm and/or bilateral pitting oedema), and 0.9% (8/884) died. Only 54.2% of the children recovered with no episode of SAM by the end of the study. Of those who developed SAM half still had MAM at the end of the follow up period. The median (interquartile range) time to recovery was 9 (4–15) weeks. Children with the lowest MUAC at enrolment had a significantly higher risk of remaining with MAM and a lower chance of recovering.

**Conclusions:**

Children with MAM during the post-harvest season in an area not eligible for SFP experience an extremely high incidence of SAM and a low recovery rate. Not having a targeted nutrition-specific intervention to address MAM in this context places children with MAM at excessive risk of adverse outcomes. Further preventive and curative approaches should urgently be considered.

## Introduction

Acute malnutrition continues to be a major global public health problem, affecting an estimated 51.5 million children aged under 5 years and being associated with at least 12.6% of all under-5 child mortality [1]. This figure is likely to be an underestimate given the burden of acute malnutrition is better estimated using incidence figures rather than prevalence alone. Moderate acute malnutrition (MAM) is more prevalent than severe acute malnutrition (SAM) and affects approximately 64% of all those categorized as having acute malnutrition [1]. Children with MAM do not yet display the same degree of wasting and other clinical complications as those with SAM, although the causes are thought to be similar. However, patients with MAM are also in a highly vulnerable state and need to be treated before their condition progresses to SAM. Compared to well-nourished children, children with MAM have a three-fold increased risk of mortality, increased risk of infections and impaired physical and cognitive development [2].

Whilst the contribution of SAM to global mortality and morbidity has been widely recognized [3,4] with resulting international treatment protocols [5], the management of MAM remains debated [6–8]. Despite much effort in recent years to harmonise the understanding of MAM and its management [9,10], consensus is still not reached, especially in non-emergency and relatively food secure settings of low and middle income countries [6,7]. Targeted Supplementary Feeding Programmes (TSFPs) are the most commonly used approach for treating MAM [11]. They use a variety of different products, including fortified blended flours and ready-to-use supplementary food (RUSF) supplements [12]. The World Health Organization has issued guidance on the recommended nutrient composition of such supplements [13] and the effectiveness of some of these different products have been compared in recent reviews [12,14]. There are other approaches being assessed for their effectiveness in the prevention and treatment of MAM, delivered both with and without nutritional supplements. For example, some studies have suggested that nutrition counselling, particularly focusing on improving infant and young child feeding practices, may be as effective as specialized food-based interventions for the treatment of MAM [15–17]. Other approaches aim to increase dietary diversity from existing sources of natural foods, without using supplements or replacing usual family meals [18]. Current strategies of preventing MAM are even more diverse, tackling the underlying causes of undernutrition through a combination of food security, behaviour change, water and sanitation, medical, cash-based, and surveillance approaches [11,19–21]. There is an incomplete evidence base for their effectiveness [7].

In Ethiopia, acute malnutrition remains an extensive and seemingly embedded problem. During the Demographic and Health Survey of 2011, 10% of children aged under-five in the country had acute malnutrition, with 70.0% of those having MAM [22]. The current strategy in Ethiopia is to restrict SFPs for treatment of MAM to selected woredas (districts) of the country defined as chronically food insecure. In areas not considered chronically food insecure there are no food supplementation programmes, and instead there is reliance on existing strategies such as the Enhanced Outreach Strategy (EOS) which delivers vitamin A supplementation and deworming, water treatment, improved sanitation and nutrition counselling. However, it is increasingly recognised that relative food security at the woreda level does not necessarily equate to nutritional security in all households [22–25]. Furthermore, children with MAM require food of sufficient energy and nutrient density to recover [26]. A recent unpublished survey in nineteen food secure woredas of Ethiopia revealed an average prevalence of MAM of 4.9%, which represents an alarming burden at the national scale. The paucity of data describing the short-term outcomes of children who have MAM in food secure areas with no food supplementation programme, alongside the corresponding need for evidence-based policy recommendations, prompted the design of this prospective observational study.

## Materials and Methods

### Study design

This was an observational, prospective cohort study of children aged 6–59 months with MAM living in a setting without SFP provision, followed weekly for seven months. Our primary objectives were to determine the proportion of recovery, non-response and deterioration to SAM; the incidence of mortality and SAM, and the average duration of the MAM episode. Our secondary objective was to identify child, household, and caretaker characteristics associated with the different outcomes and to compare the outcomes with reports from SFPs in food-insecure settings.

**Fig 1:**
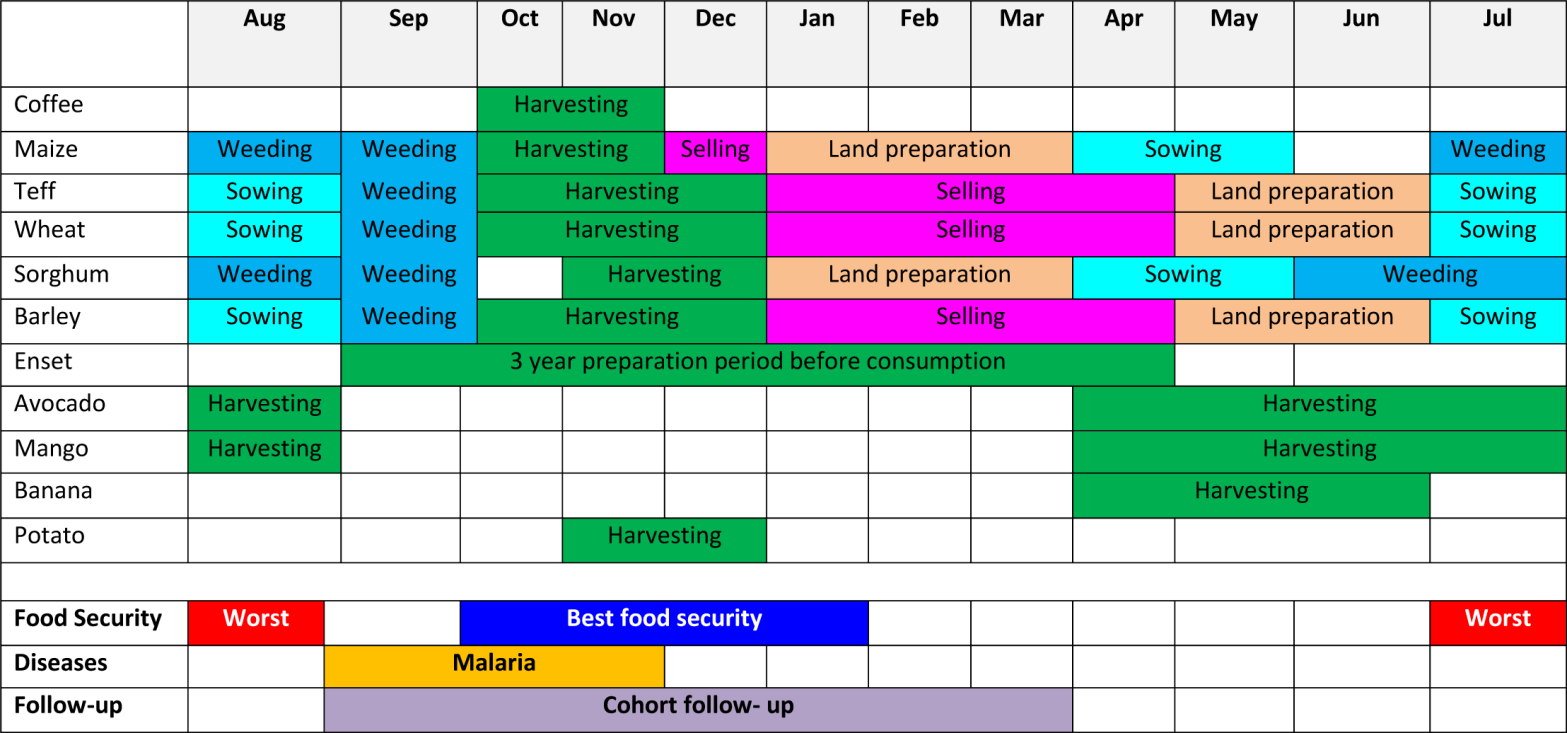
Agricultural calendar of Jimma zone and study implementation period.

MAM was defined as having mid-upper arm circumference (MUAC) of ≥11.0cm and <12.5cm with no bilateral pitting oedema. This definition comprises an expanded MUAC bracket to incorporate two cut-offs; the in-country criteria and the World Health Organisation (WHO) 2009 criteria. The Ethiopian Emergency Nutrition Coordination Unit (ENCU) defines MAM as MUAC ≥11.0cm and <12.0cm [27], whereas the WHO (2009) define MAM as MUAC ≥11.5cm and <12.5cm [28]. We therefore decided to use the expanded bracket in order to collect data that would be applicable to both the Ethiopian and international nutrition communities. Unless otherwise specified the definition of SAM in our main results section uses the ENCU definition of MUAC <11.0cm and/or bilateral pitting oedema [27]. The period of follow-up was set at 7 months (September 2013 –March 2014) in order to capture data from the end of the peak malnutrition period right through to the post-harvest period. The agricultural calendar for Jimma Zone indicating the study implementation period is shown in Fig 1.

### Study setting

The study was conducted in Mana and Dedo woredas of Jimma Zone in South-western Ethiopia. These study areas were selected to represent rural settings with no access to SFPs, but where other health and nutrition services were delivered according to the national policy. These services included the Integrated Management of Maternal, Neonatal and Childhood Illnesses (IMNCI), the Integrated Community Case Management of illness (ICCM), and the Community-based Management of Acute Malnutrition (CMAM) to treat SAM. Through the Enhanced Outreach Strategy (EOS) vitamin A supplementation and deworming treatment was scheduled for distribution at 6-monthly campaigns. Immunisation, basic nutrition and sanitation counselling were provided through the health extension system. Subsistence farming is the dominant form of livelihood in the area. Major crops produced in the area are cereals (maize, teff, sorghum and barley), pulses (beans and peas), cash crops (coffee and khat), and root crops (false banana and potato) [29]. Mana and Dedo woredas have a similar agro-ecological pattern. Despite being known as one of the more food secure regions in Ethiopia the area experiences seasonal deterioration of food security.

### Sample Size

The sample size for the expected number of children deteriorating to SAM was calculated using the web-based OpenEpi software [30]. Anticipating a 10% deterioration to SAM with a 95% confidence level of ±2.5%, assuming a design effect of 1.5 and a loss to follow up of 20%, the required sample size was 996. The choice of 10% was based on the observed deterioration to SAM in a cohort of MAM children enrolled in a supplementary feeding programme in two regions of Ethiopia [31]. The sample size calculated for the proportion of recovered children was 691 children. This was calculated using the same loss to follow up proportion and design effect but assuming a 49± 5% recovery rate as in the evaluation mentioned above [31]. The target sample size of 996 was not reached but the loss to follow up was much lower than anticipated, making our sample size sufficient for the primary and secondary outcomes.

### Enrolment into study, follow up and definition of outcomes

**Fig 2:**
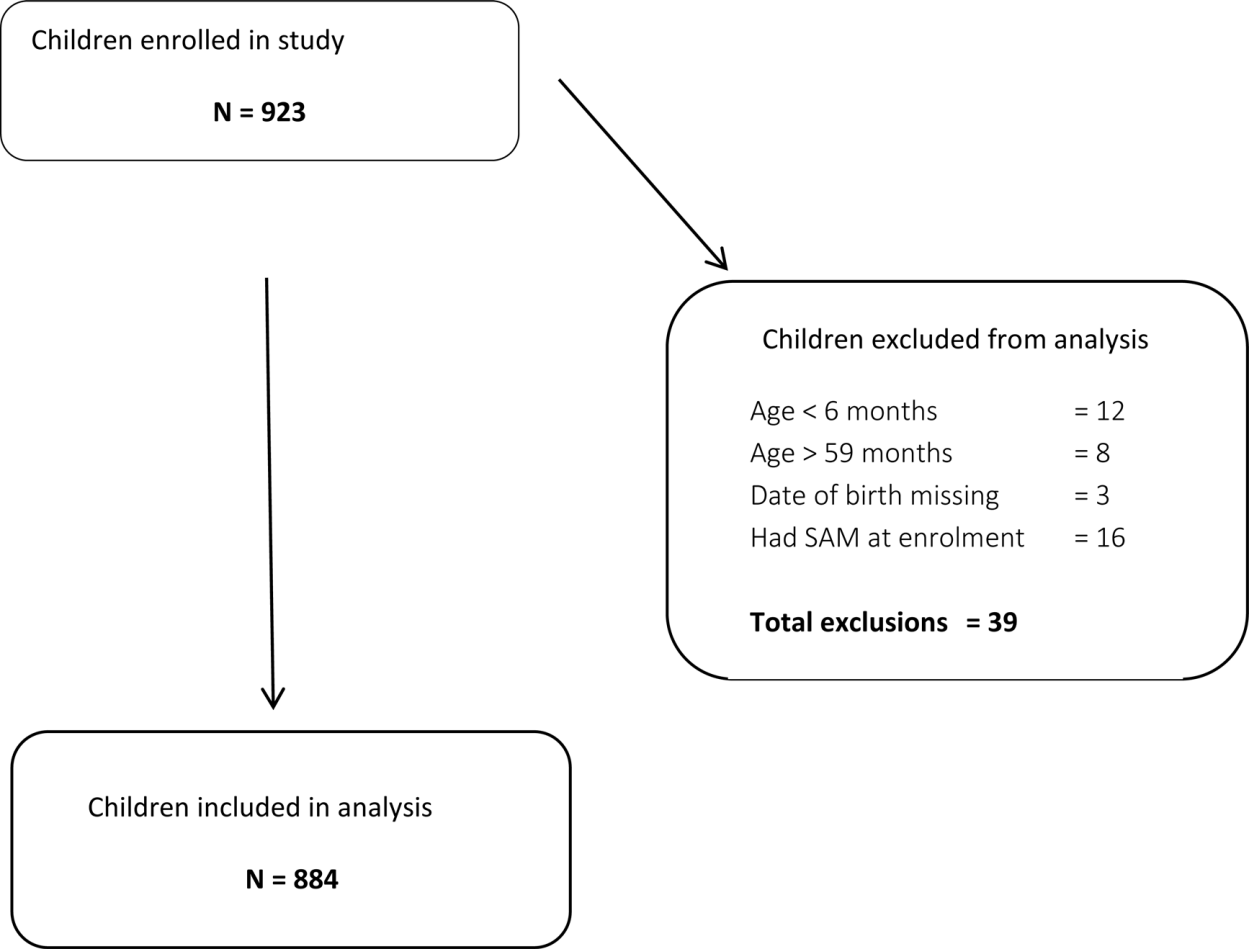
Subject flow diagram.

Children were identified during a 10-day (5 days per woreda) exhaustive house-to-house MUAC and bilateral pitting oedema screening implemented by 82 trained community health volunteers (CHVs) in August-September 2013. Cohorts in Mana and Dedo woredas were started within two weeks of each other. During the initial screening 923 children were identified as eligible after determining the child’s MUAC met the MAM definition with no bilateral pitting oedema or medical complications and was not planning to move out of the study area. Forty data collectors then collected the baseline household questionnaire within the following week and performed weekly home visits for the subsequent 28 weeks. Fig 2 summarises the flow of participants through the study resulting in 884 children included for analysis.

Final outcomes were assigned to children after 28 weeks of follow up. ‘Recovered’ was defined when a child obtained MUAC ≥12.5cm with no oedema by week 28 and had never experienced an episode of SAM throughout the follow up. ‘Deteriorated to SAM’ was defined as reaching a MUAC <11.0 cm or presenting bilateral pitting oedema any time during follow up. ‘Died’ was a death recorded during the study follow up, confirmed by a home visit. ‘Loss to follow up’ were those children who dropped out of the study due to moving away or refusal. ‘Remained MAM’ was defined as a child who never experienced a SAM episode but whose MUAC still remained below 12.5cm at the 28th week of follow up. We further categorised these children into those who remained with MAM throughout the entire follow up period, and those who relapsed to MAM. The relapsed children were defined as those who had reached the recovery criteria of MUAC ≥12.5cm for two consecutive weeks but deteriorated back to MAM before the 28^th^ week of follow up.

### Data collection methods

We designed a baseline questionnaire to capture potential predictors of the children’s final outcomes. The questionnaire included child, household and caretaker related variables. All predictors of outcomes are fully defined in the following section. Child-related variables included sex, age at enrolment, feeding index, immunization status, access to EOS, common illnesses in the previous two weeks (diarrhoea, fever, cough, difficulty breathing), hand washing practices, bed net use and baseline weight, height and MUAC. Household variables included questions to assess wealth index, household size, main income generating activity, food security status, water and sanitation questions, geographical access to primary health care, death of a family member and household head information. Caretaker-related variables included relationship to the child, age, educational status, occupation, work burden index, access to and source of information about recommended child feeding and care practices, hand washing practices, disposal of young child faeces, health seeking behaviour and MUAC. Weekly questionnaires were designed to track the cohort’s anthropometric, mortality and morbidity profile over the follow up period. We obtained information on common childhood illnesses, weight, MUAC and development of nutritional oedema. Height was taken monthly. Both the baseline and weekly questionnaires were pre-tested and translated into Amharic and Afan Oromo languages.

Nutritional oedema was assessed by pressing the thumbs down on both feet, holding for three seconds and observing if any bilateral indentation remained. Weight was measured using SECA 874 digital floor scales to the nearest 50 grams. Weight of small children was taken together with their caretakers. Height was measured for children aged ≥2 years and length for children <2 years. Both measures were taken using locally produced wooden boards to the nearest 0.1cm. MUAC was measured on the left arm using standard numbered insertion tapes to the nearest 0.1cm. The anthropometric measures were taken of the children in minimal clothing and without shoes using standard measurement techniques [32]. Immunisation status was only recorded for children with an immunisation record card.

As measures of child morbidity we asked the caretaker if the child had experienced diarrhoea, fever, cough or difficulty breathing in the prior two weeks. Diarrhoea was defined as 3 or more loose stools per day and fever as being hot to the touch.

The household wealth index was assessed using the standard criteria from the Ethiopia 2011 Demographic and Health Survey [22], which included a durable asset list, recording the land and animals owned and observation of housing materials. Healthcare accessibility was captured by recording the transportation used to reach the nearest health facility and the time taken to reach there.

To minimise bias, all data collectors received a standardised training for conducting the anthropometry and questionnaires. CHVs received a one-day refresher on MUAC screening techniques, and data collectors received 8 days of training on anthropometry and questionnaire delivery. The study was preceded by pilot testing all of the data collection tools, conducted within the study area but in households not selected for the study. The data collectors were closely supervised to confirm adherence to the interview procedures.

### Creation of predictor variables

Anthropometric indices (weight-for-height, height-for-age and weight-for-age) were calculated in Stata according to WHO (2006) growth standards [33] using the zscore06 command [34]. Weight-for-height z-score (WHZ), height-for-age z-score (HAZ) and weight-for-age z-score (WAZ) at enrolment were categorised into <-3 z-scores, ≥-3 and <-2 z-scores and >-2 z-scores. Child MUAC was split into 11.0–11.4cm, 11.5–11.9cm and 12.0–12.4cm categories. Caretaker MUAC was categorised into MUAC <23cm and ≥23cm.

A respiratory illness variable was created and 3 categories defined: those having no cough, those with cough but no difficulty breathing and those with cough and difficulty breathing.

The feeding practice of children was summarised using the Infant and Child Feeding Index (ICFI) score [35]. The ICFI creates three categories based on a final score derived from the caretaker’s previous day recall of breastfeeding, dietary diversity in 7 food groups as per methodology in Arimond and Ruel [36] and feeding frequency [37]. The scores are assigned according to the child’s age category, with each age category scoring a maximum of 6 (S1 Table). The final three ICFI categories were then grouped so that those with scores of 0–2 were assigned to the lowest group, 3–4 to the middle group and 5–6 to the highest group.

Hand washing scores for the child and caretaker were created by assigning no = “0” and yes = “1” for seven hand washing questions, totalling the scores and dividing into three categories with the lowest category containing scores 0–2, the middle one containing scores 3–5 and the highest category containing scores 6 and 7. Hand washing questions for the caregiver were for after defecation, before preparing food, before breastfeeding, after cleaning child’s faeces, before eating, after eating and when hands are dirty. Hand washing questions for the child were regarding before and after defecation, before eating, after eating, after crawling and after touching dirty things.

The caretaker’s work index was created using responses to questions on how many days in the preceding week the caretaker had worked on herding animals, farm work, fetching water, washing clothes, cooking, child care, grinding grain and carrying a heavy load. The answers were summed and divided into tertiles to form ‘least heavy’, ‘middle’ and ‘most heavy’ categories.

Wealth index scores and resulting quintiles were generated from the data collected on wealth using the criteria listed above by principal components analysis using the methodology described in detail in Vyas and Kumaranayake [38].

Healthcare accessibility categories were defined based on the time taken to reach the nearest health facility as ≤30 minutes, >30–60 minutes and >60 minutes. The number of different topics covered in counselling sessions from a health extension worker in the past month was summarised in three categories: none received, 1–2 topics, and 3 or more topics. Caretakers’ knowledge of danger signs was summarised into three categories: zero, 1–3, and 4 or more signs (out of a total of 7 possible answers).

Two health-seeking behaviour variables were created regarding what the caregiver did when the child was ill. ‘Good’ health-seeking behaviours were defined as taking the child to the health facility, giving the child modern medicine, giving more food, giving more clean water, having a separate plate for the child and increasing the amount attention given. ‘Poor’ behaviours included giving traditional medicine, giving less food and water, and prioritising a traditional healer or other faith healing practices.

Water, sanitation and hygiene (WASH) questions were summarised as recommended by the WHO & UNICEF (2006) methodology [39], producing the standard binary variables of improved/unimproved drinking water sources and latrine type, and safe/unsafe water treatment and disposal of youngest child’s faeces.

Household food security status was summarised using the nine questions and scoring methodology from the Household Food Insecurity Access Scale (HFIAS), described in detail by Coates *et al*. [40].

### Data analysis

We described baseline characteristics of the cohort using proportions for categorical variables, means for normally distributed continuous variables and medians for non-normal continuous variables. For our main analysis we present results using the MAM and SAM definitions as per our study design, using Ethiopian government cut-offs. However, in order to also present data that can be externally validated using WHO (2009) cut-offs, we also analysed our primary objectives using a subsample of our cohort. Here we defined SAM as MUAC <11.5cm and MAM as MUAC 11.5–12.4cm, therefore excluding children enrolled with MUAC 11.0–11.4cm (n = 111).

We calculated deterioration to SAM incidence using the first episode only. Since deterioration to SAM was the primary outcome, we listed any child who experienced an episode of SAM under that category, and listed any further details as sub-categories (*i*.*e*. those who had SAM who then recovered, became MAM, died, or were lost to follow up).

We stratified Kaplan-Meier survival plots for deterioration to SAM by MUAC admission category and compared curves using the log-rank test with Bonferroni correction in the case of multiple comparisons. To analyse potential predictors for the outcomes we performed univariable and multivariable Cox regression for deterioration to SAM and recovery, and multinomial univariable and multivariable logistic regression for the ‘remaining MAM’ outcomes. In the case of the latter we included two levels in our multinomial outcome: children who recovered but then relapsed before the 28^th^ week of follow up, and those who remained MAM throughout the follow up. These outcomes were compared against children who recovered. Children who died and were lost to follow up were excluded from our multinomial logistic regression models. The models provided relative risk ratios and their 95% confidence intervals.

For the cox regression analyses we checked whether proportional hazards assumptions were met using three methods. Firstly, we inspected the -ln(-n(survival)) versus ln(analysis time) plots (stphplot command in Stata) to look at convergence of curves. Secondly, we used the smoothed scaled Schoenfeld residuals versus time plots to test for a non-zero slope (estat phtest Stata command). Thirdly, we plotted a lowess curve to assess how much the scaled Schoenfeld residuals versus time plots deviated from the horizontal reference line at y = 0 (stphtest Stata command). The proportional hazards ratio assumptions were not met for some explanatory variables (MUAC category, gender and HAZ category) and so we performed extended Cox proportional hazards models that took into account the non-proportionality of these variables. Since the results of the extended models had little influence on the interpretation of the results the simpler models (with proportional hazards violations) were reported for ease of interpretation, as suggested by Allison (1995)[41]. For completeness we present the extended models in Tables A-C of S1 File, which include details on the time*covariate interaction terms fitted for the violating variables.

Given that our goal was to identify primary predictors whilst controlling for putative confounding variables, variable selection was done manually using the p-value and the change-in-estimate method [42,43]. Robust standard errors were used throughout. No imputation method for missing values was used. We restricted all the analysis to children with complete information. Data were double entered at the field level using Epi Data version 3.2 [44]. Data cleaning and all data analyses were performed using Stata 13.0 [45].

### Ethical considerations

The study was approved by the ethical review board of Jimma University (reference RPGC/131/2013). Enrolment into the study was voluntary and data collection was initiated after obtaining written consent or thumbprint from the caregiver of the child. Any child who deteriorated to SAM (MUAC <11.0cm or bilateral pitting oedema) at any point during the follow up was referred to the nearest health facility for outpatient therapeutic feeding programmes as per existing national protocol, and continued to be followed up.

## Results

### Baseline characteristics

Table 1 summarises the socio-demographic characteristics, WASH practices, food security situation, and health and nutrition conditions at baseline of the 884 children analysed. Our cohort was relatively young and had more girls than boys. Mothers were also young with the majority of them having no formal education. Most houses did not safely treat their drinking water, and significant proportions did not have improved sanitation facilities or drinking water sources. EOS and Health Extension Worker (HEW) activities were sub-optimal as indicated by the proportion of those who did not receive a HEW visit in the past month, alongside the low coverage of those who received preventative vitamin A supplementation and deworming medication in the past 6 months. Less than a quarter of children were sleeping under insecticide-impregnated mosquito nets. The majority of households were categorised as having food insecurity, with a third of them experiencing severe food insecurity. Over two thirds of children were stunted (HAZ <-2) at enrolment with almost half of children being already severely stunted (HAZ <-3). Over 60% of children were not wasted (WHZ ≥-2). The morbidity was high as indicated by the proportion of children who experienced diarrhoea and cough in the past two weeks.

**Table 1:** Baseline household and child characteristics.

Variable *(statistics description)*	Statistics	Total N
Child sex: male, *n (%)*	360 (40.9)	881
Child age (months), *median (IQR)*	12 (12–34)	884
Household size, *median (IQR)*	6 (5–7)	884
Primary caretaker is mother, *n (%)*	826 (93.4)	884
Caretaker education: no formal, *n (%)*	648 (73.3)	884
Caretaker age, *median (IQR)*	30 (25–35)	884
Mother's MUAC category: <23cm, *n (%)*	615 (69.6)	884
Sex of the household head: male, *n (%)*	818 (92.8)	881
Household head education: no formal, *n (%)*	518 (58.6)	884
Number of people per sleeping room, *median (IQR)*	4 (3–6)	880
Person died in the household in past year: yes, *n (%)*	71 (8.0)	884
Number of food groups eaten in past 24 hours (child), *median (IQR)*	2 (1–3)	884
Household food security		
Food secure, *n (%)*	288 (32.6)	884
Mildly food insecure, *n (%)*	58 (6.6)	884
Moderately food insecure, *n (%)*	24 (27.3)	884
Severely food insecure, *n (%)*	297 (33.6)	884
Drinking water source: unimproved, *n (%)*	294 (33.3)	884
Drinking water treatment: unsafe, *n (%)*	869 (98.3)	884
Sanitation facility: unimproved, *n (%)*	136 (15.4)	884
Faeces disposal: unsafe, *n (%)*	246 (27.8)	884
Service from health extension worker received in past month: none, *n (%)*	264 (29.9)	884
Vitamin A received in past 6 months: yes, *n (%)*	610 (69.0)	884
Deworming received in past 6 months (children aged >24mo): yes, *n (%)*	191 (52.2)	366
Child sleeps under a bed net: yes, *n (%)*	189 (21.4)	882
Transportation to nearest health facility: on foot, *n (%)*	836 (94.6)	884
Diarrhoea in past 2 weeks: yes, *n (%)*	28.3 (28.4)	884
Fever in past 2 weeks: yes, *n (%)*	265 (30.1)	882
Cough in past 2 weeks: yes, *n (%)*	249 (28.2)	882
Breastfed (age 6–24 months): yes, *n (%)*	468 (90.3)	518
WHZ, *median (IQR)*	-1.7 (-2.4,-1.0)	872
<-3, *n (%)*	111 (12.7)	872
≥-3 and <-2, *n (%)*	219 (25.1)	872
≥-2, *n (%)*	542 (62.2)	872
HAZ, *median (IQR)*	-2.9 (-4.4, -1.6)	880
<-3, *n (%)*	416 (47.3)	880
≥-3 and <-2, *n (%)*	176 (20.0)	880
≥-2, *n (%)*	288 (33.7)	880
WAZ, *median (IQR)*	-2.8 (-3.7, -1.9)	873
<-3, *n (%)*	400 (45.8)	873
≥-3 and <-2, *n (%)*	230 (26.3)	873
≥-*2*, *n (%)*	243 (27.8)	873

Abbreviations: IQR, interquartile range; MUAC, mid-upper arm circumference; WHZ, weight-for-height z-score; HAZ, height-for-age z-score; WAZ, weight-for-age z-score.

### Final outcomes of the MAM cohort

Table 2 gives the percentage of children who recovered with no episode of SAM, of those who remained with MAM, of those who experienced at least one episode of SAM, of those who died without experiencing SAM and of those who were lost to follow by the end of the study. Of those who developed SAM half still had MAM at the end of the follow up. Of the 32 children lost to follow up with no episode of SAM, 23 (71.9%) were MAM status at the last known measurement and 9 (28.1%) had MUAC ≥12.5cm.

**Table 2:** Final Outcomes of the MAM cohort after 28 weeks of follow up.

	All children		Children with SAM or MAM outcomes	
Outcome	n	%	n	%
Recovered and never SAM	479	54.2		
Remained MAM (never SAM)	287	32.5		100.0
Remained MAM during the entire follow up			192	66.9
Recovered during follow up but relapsed to MAM			95	33.1
Total SAM (at least 1 episode)	82	9.3		100.0
SAM at week 28			9	11.0
SAM at least once but recovered			27	32.9
SAM at least once but MAM at week 28			41	50.0
SAM at last visit before death reported			4	4.9
SAM at least once but lost to follow up			1	1.2
Lost to follow up	32	3.6		
Died (without SAM at last visit before death reported)	4	0.5		
Total	884	100.0		

Abbreviations: SAM, severe acute malnutrition; MAM, moderate acute malnutrition.

**Fig 3:**
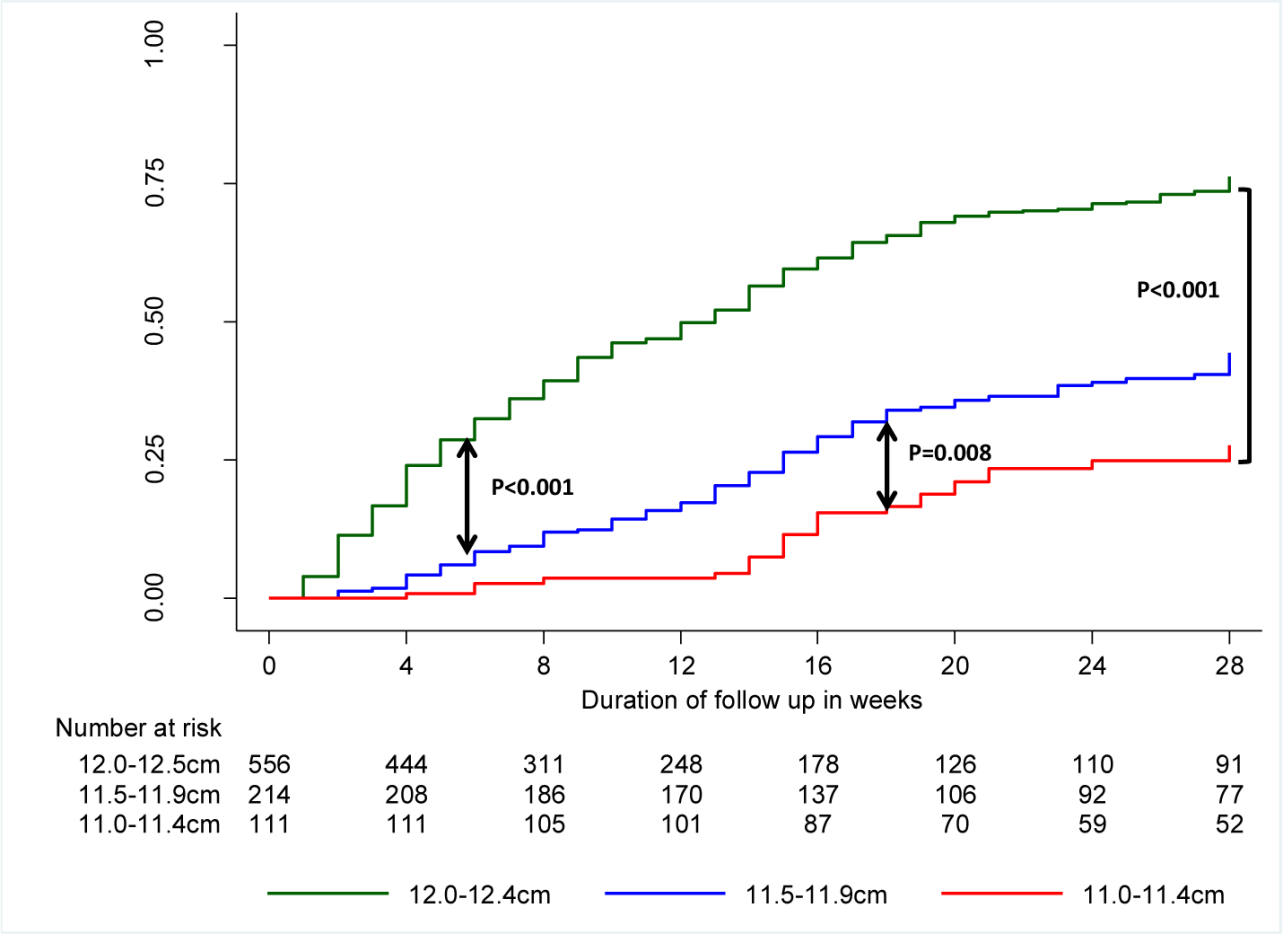
Kaplan-Meier failure function estimates for recovering from moderate acute malnutrition with no episode of severe acute malnutrition during follow up, by mid-upper arm circumference category at enrolment.

Fig 3 illustrates the probability of recovery from MAM over time stratified by MUAC enrolment category. The mean (95% CI) cumulative probability of recovering by the 28^th^ week of follow up without experiencing an episode of SAM was 65.9 (62.0–69.8)%. The median (IQR) time to recovery was 9 (4–15) weeks. These parameters varied significantly according to the category of MUAC at enrolment. The mean cumulative probability of recovering was 79.6 (75.3–83.7)% for those with MUAC at enrolment between 12.0cm and 12.4cm, 49.2 (41.0–58.1)% for those with MUAC at enrolment between 11.5cm to 11.9cm and 31.3 (21.7–43.9)% for those with MUAC at enrolment between 11.0cm and 11.4cm. Pairwise comparison shows that the difference between the survival curves for the three categories of MUAC was statistically different (log-rank test p <0.001). For those who recovered, the median times to recovery from enrolment for the same categories of MUAC were 7 (4–14) weeks, 13 (8–17) weeks and 16 (14–20) weeks, respectively. Of note, the cumulative probability of recovery increases steeply from enrolment in children enrolled with MUAC between 12.0cm and 12.4 cm, indicating a faster recovery rate for this category. The figure also shows that for children enrolled with MUAC between 11.0cm and 11.4cm, most of the recovery occurred between the 12^th^ and 20^th^ weeks. For all three categories there was almost no more recovery after the 20^th^ week of follow up.

The incidence of death was 0.13 per 100 person-months, equating to an under-5 mortality rate of 0.44 deaths per 10,000 child days. Of the 8 children who died, 7 had a MUAC <11.5 cm at one point before the death was recorded, including 3 who had a MUAC <11.0cm. Four children were in an episode of SAM the week before they died while 4 were in an episode of MAM as per the Ethiopian operational definition of MUAC>11.0cm. The timing of death was spread out over the duration of the follow up with the last visit varying between weeks 3 and 27 of follow up. All caregivers attributed the deaths to disease rather than accident.

Overall the incidence rate of SAM was 1.57 per 100 children-months. This figure varies according to the MUAC category at enrolment. When compared to children enrolled with a MUAC between 12.0cm and 12.4cm, the incidence of SAM was 3.8 and 14.9 times higher in children enrolled with a MUAC between 11.5 cm and 11.9 cm and between 11.0 cm and 11.4 cm respectively (Table 3).

**Table 3:** Incidence of Severe Acute Malnutrition as per Ethiopian operational definition and protocols (MUAC <11cm or bilateral pitting oedema).

Stratification by MUAC at enrolment	N	No. of episodes	Follow-up time (100 months)†	Incidence rate (95% CI) per 100 person-months‡	Incidence rate ratio (95% CI)
All children	884	82	52.3	1.57 (1.26–1.94)	
12.0 - <12.5 cm	558	18	34.7	0.52 (0.32–0.80)	1.0
11.5 - <12.0 cm	215	25	12.6	1.99 (1.32–2.89)	3.8 (2.0–7.4)
11.0 - <11.5 cm	111	39	5.03	7.75 (5.59–10.5)	14.9 (8.3–27.7)

† Total time at risk, expressed in units of 100 person-months.

‡ Incidence of first episode of SAM, expressed per 100 person-months.

Abbreviations: MUAC, mid-upper arm circumference; CI, confidence interval.

**Fig 4:**
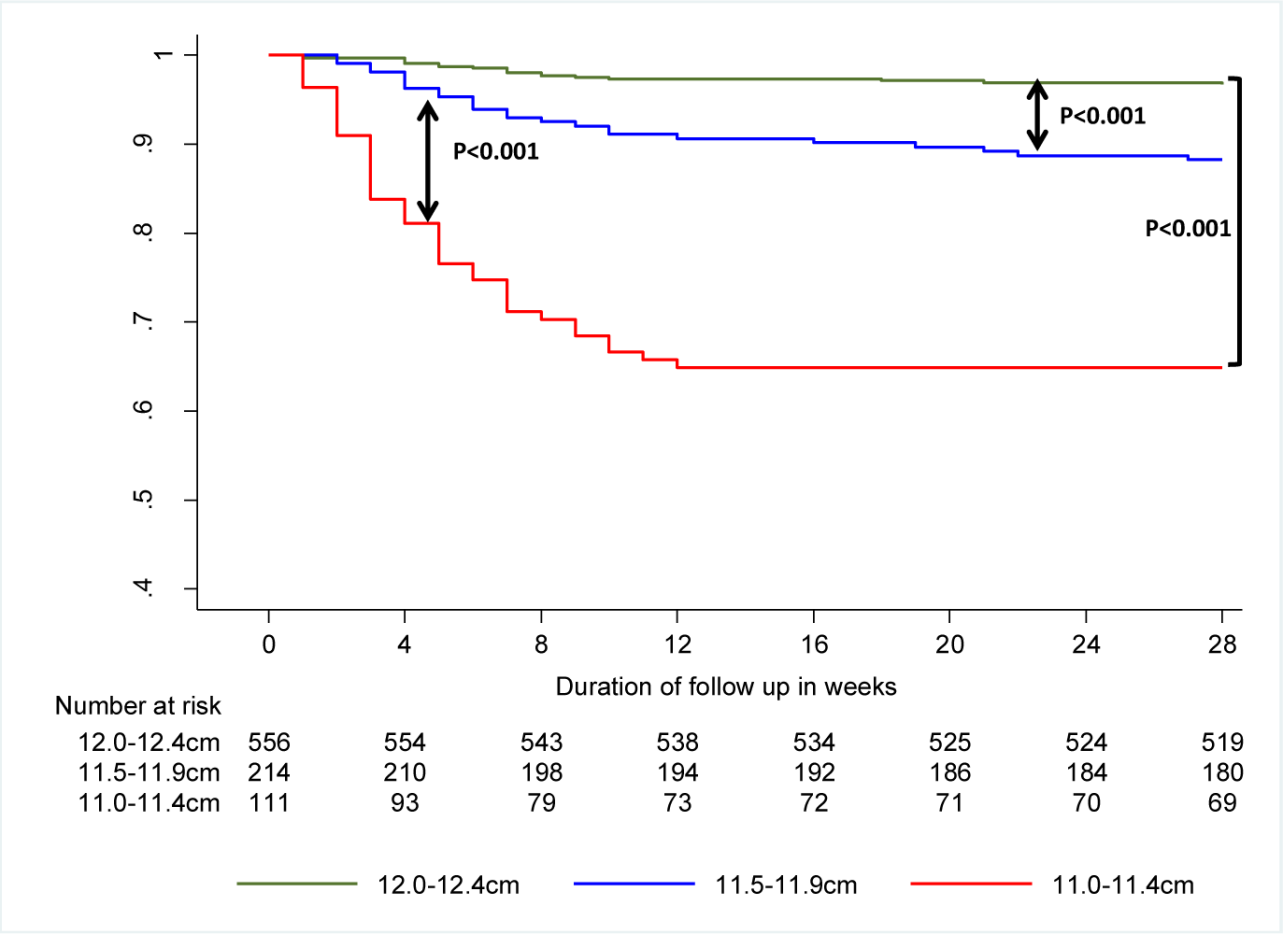
Survival curve for developing Severe Acute Malnutrition as per Ethiopian operational criteria (MUAC <11cm or bilateral pitting oedema), stratified by MUAC category at enrolment.

Fig 4 indicates the cumulative probability of children not deteriorating to SAM over the follow-up period. Overall, the 28-weeks cumulative probability of not developing SAM was 90.5 (84.2–92.3)%. However, this varied considerably by category of MUAC at enrolment, being 96.5 (94.5–97.8)% for children enrolled with MUAC 12.0–12.4cm, 88.2 (83.1–91.9)% for children enrolled with MUAC 11.5–11.9cm and only 64.9 (55.2–72.9)% for those enrolled with MUAC 11.0–11.4cm (test for trend for survival functions; p<0.001). The sharpest drop of probability was during the first 12 weeks of follow up, thereafter dropping little.

### Predictors of outcomes

Table 4 reports the list of predictors of deterioration to SAM retained. MUAC at admission was the only variable independently associated with risk of developing SAM during follow up (p<0.001) in both the standard Cox proportional hazard analysis (Table 4) and the extended Cox proportional hazard analysis (Table A of S1 File).

**Table 4:** Predictors of occurrence of severe acute malnutrition based on parameters at enrolment: bivariate and Cox proportional hazards analyses^†^.

Variable	Category	N‡	% SAM	Unadjusted HR (95% C.I.)	P-value	Adjusted HR (95% C.I.)	P-value
Gender	Male	360	9.4	1.00 (base)		1.00 (base)	
	Female	521	9.2	0.96 (0.62–1.49)	0.859	0.98 (0.61–1.57)	0.937
Age group	≥ 24 months	366	7.9	1.00 (base)		1.00 (base)	
	< 24 months	518	10.2	1.30 (0.83–2.04)	0.255	1.32 (0.79–2.20)	0.292
Food insecurity	None	288	7.6	1.00 (base)		1.00 (base)	
	Moderate	299	9.0	1.18 (0.67–2.08)	0.557	1.33 (0.75–2.36)	0.335
	Severe	297	11.1	1.49 (0.87–2.56)	0.146	1.37 (0.78–2.39)	0.273
Child feeding practices score tertiles	Lowest	175	8.6	1.00 (base)		1.00 (base)	
	Middle	384	9.1	1.10 (0.58–1.96)	0.828	1.33 (0.71–2.52)	0.374
	Highest	325	9.8	1.14 (0.62–2.11)	0.667	1.58 (0.83–3.01)	0.161
MUAC category^§^	12.0–12.4 cm	558	3.2	1.00 (base)		1.00 (base)	
	11.5–11.9 cm	215	11.6	3.73 (2.03–6.84)	<0.001	3.60 (1.91–6.80)	<0.001
	11.0–11.4 cm	111	35.1	13.5 (7.69–23.53)	<0.001	12.31(6.63–22.85)	<0.001
WHZ category (Z-score)	≥-2	540	6.7	1.00 (base)		1.00 (base)	
	≥-3 and <-2	219	12.3	1.92 (1.16–3.16)	0.002	1.35 (0.80–2.38)	0.308
	<-3	111	15.3	2.48 (1.39–4.42)	0.011	1.37 (0.74–2.53)	0.255
HAZ category (Z-score)^§^	≥-2	288	7.3	1.00 (base)		1.00 (base)	
	≥-3 and <-2	176	6.8	0.93 (0.46–1.89)	0.089	0.88 (0.42–1.84)	0.738
	<-3	416	11.5	1.60 (0.96–2.67)	0.072	1.38 (0.78–2.44)	0.270
Topics covered by the HEW counselling	≥ 3	145	6.2	1.00 (base)		1.00 (base)	
	1–2	207	6.3	1.03 (0.44–2.41)	0.941	0.89 (0.38–2.10)	0.789
	0	531	11.3	1.88 (0.93–3.78)	0.078	1.63 (0.80–3.32)	0.181
Vitamin A in past 6 months	No	274	12.0	1.00 (base)		1.00 (base)	
	Yes	610	8.0	0.65 (0.42–1.01)	0.058	1.10 (0.69–1.76)	0.690

†N in multivariable analysis: 869 with 80 events.

‡N in bivariate analysis

§The proportional hazards assumptions were violated for these variables and the HR therefore should be interpreted as an average HR over the period of follow up.

Abbreviations: SAM, Severe Acute Malnutrition; CI, Confidence interval; HR, Hazard Ratio; MUAC, mid-upper arm circumference; WHZ, weight-for-height z score; HAZ, height-for-age z-score; HEW, health extension worker

Tables 5 and 6 present unadjusted and adjusted predictors of relapsing to MAM and remaining MAM throughout the follow up. Independent predictors of relapsing to MAM after recovering were MUAC, age and WHZ at admission. Independent predictors of remaining MAM throughout the follow up period were MUAC, WHZ at admission, food insecurity level, child feeding practices score, WASH practices (source of drinking water, child stools disposal practices), mother’s working index, household wealth quintile and self-reported intake of Vitamin A in the previous 6 months.

**Table 5:** Baseline predictors of remaining MAM at the end of follow up; unadjusted relative risk ratio and 95% CI for remaining MAM and for relapsing to MAM after recovering^†^ (n = 763).

Variable	Category	n	% Relapsed	% MAM	Recovered but relapsed to MAM compared with fully recovered		Remained MAM throughout compared with fully recovered	
					RRR (95% CI)	P-value	RRR (95% CI)	P-value
Gender	Male	309	10.4	24.3	1.00 (base)		1.00 (base)	
	Female	454	13.7	25.6	1.42 (0.89–2.26)	0.136	1.14(0.81–1.61)	0.435
Age group	≥ 24 months	324	9.3	24.4	1.00 (base)		1.00 (base)	
	< 24 months	427	14.6	25.7	1.75 (1.09–2.80)	0.019	1.17 (0.84–1.65)	0.355
MUAC category	12.0–12.4 cm	515	10.9	17.7	1.00 (base)		1.00 (base)	
	11.5–11.9 cm	179	14.0	39.7	1.98 (1.17–3.36)	0.011	3.46 (2.34–5.11)	<0.001
	11.0–11.4 cm	69	18.8	43.5	3.28 (1.59–6.77)	0.001	4.67 (2.63–8.28)	<0.001
WHZ category (Z-score)	≥-2	485	11.5	21.4	1.00 (base)		1.00 (base)	
	≥-3 and <-2	182	11.5	30.2	1.15 (0.66–1.99)	0.617	1.62 (1.09–2.40)	0.016
	<-3	89	18.0	34.8	2.21 (1.16–4.20)	0.015	2.31 (1.38–3.86)	0.001
Child feeding practices score tertiles	Highest	283	12.0	29.0	1.00 (base)		1.00 (base)	
	Middle	326	11.3	24.5	0.87 (0.52–1.44)	0.590	0.78 (0.54–1.13)	0.186
	Lowest	154	14.9	19.5	1.12 (0.62–2.00)	0.707	0.60 (0.37–0.98)	0.043
Food insecurity	None	258	12.0	19.8	1.00 (base)		1.00 (base)	
	Moderate	255	10.6	25.9	0.95(0.54–1.65)	0.846	1.40(0.92–2.15)	0.115
	Severe	250	14.4	30.0	1.47 (0.87–2.50)	0.153	1.86 (1.22–2.83)	0.004
Wealth quintile	1–4	602	13.1	27.2	1.00 (base)		1.00 (base)	
	5 (Richest)	150	9.3	16.0	0.57 (0.31–1.04)	0.068	0.47 (0.29–0.76)	0.002
Mother’s working index	Least heavy	279	11.5	28.0	1.00 (base)		1.00 (base)	
	Middle	219	12.8	30.6	1.19 (0.68–2.08)	0.520	1.13 (0.77–1.67)	0.520
	Most heavy	265	12.8	17.7	0.97 (0.58–1.65)	0.928	0.56 (0.37–0.84)	0.006
Stools disposal	Unsafe	216	10.6	35.2	1.00 (base)		1.00 (base)	
	Safe	547	13.0	21.2	1.00 (0.60–1.68)	0.990	0.50 (0.35–0.71)	<0.001
Drinking water source	Unimproved	247	8.5	35.2	1.00 (base)		1.00 (base)	
	Improved	516	14.1	20.3	1.43 (0.85–2.41)	0.181	0.50 (0.35–0.70)	<0.001
Hand washing score tertiles	Lowest	88	17.0	22.7	1.00 (base)		1.00 (base)	
	Middle	390	12.3	31.3	0.77(0.40–1.48)	0.435	1.47(0.84–2.57)	0.178
	Highest	285	10.9	17.5	0.54 (0.27–1.07)	0.076	0.65 (0.36–1.18)	0.159
Vitamin A in past 6 months	No	224	11.6	33.9	1.00 (base)		1.00 (base)	
	Yes	539	12.6	21.5	0.90 (0.55–1.48)	0.674	0.52 (0.37–0.75)	<0.001
Type of toilet	Unimproved	118	14.4	24.6	1.00 (base)		1.00 (base)	
	Improved	645	11.9	25.3	0.80 (0.45–1.44)	0.466	1.00 (0.62–1.59)	0.997
Topics covered by the HEW counselling	≥ 3	132	9.8	18.2	1.00 (base)		1.00 (base)	
	1–2	182	14.3	21.4	1.62 (0.79–2.90)	0.186	1.32 (0.74–2.35)	0.346
	0	448	12.3	28.6	1.52 (0.79–3.33)	0.208	1.91 (1.16–3.14)	0.010

†Model compares children who remained MAM in two categories with those children who recovered (children who died or lost to follow up are excluded).

Abbreviations: CI, Confidence interval; RRR, relative risk ratio; MUAC, mid-upper arm circumference; WHZ, weight-for-height z-score; HEW, health extension worker

**Table 6:** Baseline predictors of remaining MAM at the end of follow up; adjusted relative risk ratio and 95% CI for remaining MAM or relapsing to MAM after recovering^†^ (n = 763).

Variable	Category	n	% Relapsed	% MAM	Recovered but relapsed to MAM compared with fully recovered		Remained MAM throughout compared with fully recovered	
					RRR (95% CI)	P-value	RRR (95% CI)	P-value
Gender	Male	309	10.4	24.3	1.00 (base)		1.00 (base)	
	Female	454	13.7	25.6	1.43 (0.87–2.35)	0.156	1.10(0.75–1.63)	0.615
Age group	≥ 24 months	324	9.3	24.4	1.00 (base)		1.00 (base)	
	< 24 months	427	14.6	25.7	2.00 (1.20–3.35)	0.008	1.29 (0.86–1.93)	0.216
MUAC category	12.0–12.4 cm	515	10.9	17.7	1.00 (base)		1.00 (base)	
	11.5–11.9 cm	179	14.0	39.7	1.87 (1.07–3.27)	0.028	3.00 (1.93–4,65)	<0.001
	11.0–11.4 cm	69	18.8	43.5	2.77 (1.28–5.98)	0.009	3.93 (2.05–7.52)	<0.001
WHZ category (Z-score)	≥-2	485	11.5	21.4	1.00 (base)		1.00 (base)	
	≥-3 and <-2	182	11.5	30.2	1.13 (0.63–2.02)	0.680	1.64 (1.04–2.58)	0.033
	<-3	89	18.0	34.8	2.10 (1.04–4.21)	0.037	1.83 (1.01–3.34)	0.048
Child feeding practices score tertiles	Highest	283	12.0	29.0	1.00 (base)		1.00 (base)	
	Middle	326	11.3	24.5	0.79 (0.47–1.35)	0.396	0.68 (0.45–1.04)	0.074
	Lowest	154	14.9	19.5	0.84 (0.44–1.59)	0.589	0.46 (0.26–0.80)	0.006
Food insecurity	None	258	12.0	19.8	1.00 (base)		1.00 (base)	
	Moderate	255	10.6	25.9	0.99(0.54–1.82)	0.980	1.48(0.91–2.40)	0.112
	Severe	250	14.4	30.0	1.48 (0.82–2.68)	0.196	1.72 (1.05–2.82)	0.031
Wealth quintile	1–4	602	13.1	27.2	1.00 (base)		1.00 (base)	
	5 (Richest)	150	9.3	16.0	0.60 (0.31–1.15)	0.123	0.61 (0.36–1.05)	0.075
Mother’s working index	Least heavy	279	11.5	28.0	1.00 (base)		1.00 (base)	
	Middle	219	12.8	30.6	1.09 (0.60–1.99)	0.775	1.23 (0.77–1.96)	0.377
	Most heavy	265	12.8	17.7	0.89 (0.50–1.58)	0.683	0.45 (0.28–0.74)	0.001
Stools disposal	Unsafe	216	10.6	35.2	1.00 (base)		1.00 (base)	
	Safe	547	13.0	21.2	1.09 (0.62–1.93)	0.756	0.56 (0.37–0.86)	0.008
Drinking water source	Unimproved	247	8.5	35.2	1.00 (base)		1.00 (base)	
	Improved	516	14.1	20.3	1.37 (0.78–2.38)	0.261	0.52 (0.35–0.77)	0.001
Hand washing score tertiles	Lowest	88	17.0	22.7	1.00 (base)		1.00 (base)	
	Middle	390	12.3	31.3	0.81(0.39–1.68)	0.570	1.08(0.58–2.02)	0.808
	Highest	285	10.9	17.5	0.66 (0.30–1.43)	0.291	0.62 (0.31–1.22)	0.165
Vitamin A in past 6 months	No	224	11.6	33.9	1.00 (base)		1.00 (base)	
	Yes	539	12.6	21.5	1.12 (0.65–1.93)	0.674	0.85 (0.56–1.29)	0.450
Type of toilet	Unimproved	118	14.4	24.6	1.00 (base)		1.00 (base)	
	Improved	645	11.9	25.3	0.93 (0.49–1.75)	0.815	1.31 (0.76–2.25)	0.323
Topics covered by the HEW counselling	≥ 3	132	9.8	18.2	1.00 (base)		1.00 (base)	
	1–2	182	14.3	21.4	1.31 (0.61–2.84)	0.486	0.94 (0.48–1.82)	0.855
	0	448	12.3	28.6	1.22 (0.60–2.46)	0.584	1.29 (0.73–2.28)	0.375

†Model compares children who remained MAM in two categories with those children who recovered (children who died or lost to follow up are excluded).

Abbreviations: CI, confidence interval; RRR, relative risk ratio; MUAC, mid-upper arm circumference; WHZ, weight-for-height z-score; HEW, health extension worker

Table 7 shows the independent predictors of recovery as identified by the multivariate standard Cox proportional analysis. The indicators of wasting (MUAC and WHZ) had the strongest associations with recovery, with the most wasted children having the lowest chance of recovering by the end of follow up. Children age <24 months at enrolment had a lower chance of recovery than older children. Children of mothers with the heaviest working index category had a better chance of recovery than those of mothers of the least heavy working index category. Being in the wealthiest category was associated with an increased chance of recovery. Amongst WASH indicators, drinking water from an improved water source was associated with increased chance of recovery. The extended Cox proportional hazards analysis identified the same variables as independent predictors (Table B of S1 File). No indicator of morbidity or infant feeding practices was retained in either of the multivariable models.

**Table 7:** Baseline predictors of recovery: bivariate and Cox proportional hazards analyses^†^.

Variable	Category	N‡	% recovery	Unadjusted HR (95% CI)	P-value	Adjusted HR (95% CI)	P-value
Gender	Male	340	59.4	1.00 (base)		1.00 (base)	
	Female	500	55.0	0.89 (0.75–1.07)	0.231	0.84 (0.70–1.02)	0.083
Age group	≥ 24 months	469	60.3	1.00 (base)		1.00 (base)	
	< 24 months	374	52.4	0.88 (0.74–1.06)	0.196	0.78 (0.64–0.96)	0.020
MUAC category^§^	12.0–12.4 cm	532	69.6	1.00 (base)		1.00 (base)	
	11.5–11.9 cm	203	40.9	0.37 (0.29–0.47)	<0.001	0.40 (0.31–0.52)	<0.001
	11.0–11.4 cm	108	24.1	0.20 (0.13–0.29)	<0.001	0.24 (0.16–0.36)	<0.001
WHZ category^§^	≥-2	519	62.6	1.00 (base)		1.00 (base)	
	≥-3 and <-2	208	51.0	0.65 (0.52–0.81)	<0.001	0.70 (0.56–0.88)	0.003
	<-3	105	40.0	0.52 (0.37–0.71)	<0.001	0.69 (0.49–0.98)	0.036
HAZ category	≥-2	274	56.6	1.00 (base)		1.00 (base)	
	≥-3 and <-2	172	65.7	1.15 (0.91–1.47)	0.238	1.14 (0.89–1.48)	0.298
	<-3	394	53.0	0.80 (0.65–0.99)	0.038	0.89 (0.71–1.13)	0.351
Food insecurity	None	282	62.9	1.00 (base)		1.00 (base)	
	Moderate	281	58.4	0.88 (0.71–1.09)	0.251	0.87 (0.70–1.09)	0.228
	Severe	282	49.3	0.71 (0.57–0.89)	0.003	0.81 (0.63–1.03)	0.085
Wealth quintile	1–4	666	54.0	1.00 (base)		1.00 (base)	
	5 (Richest)	164	68.9	1.53 (1.24–1.90)	<0.001	1.34 (1.07–1.68)	0.010
Mother’s working index	Least heavy	312	54.2	1.00 (base)		1.00 (base)	
	Middle	244	51.6	0.95 (0.76–1.20)	0.673	0.97 (0.76–1.24)	0.825
	Most heavy	287	64.1	1.45 (1.18–1.79)	<0.001	1.75 (1.40–2.18)	<0.001
Drinking water source	Unimproved	277	50.2	1.00 (base)		1.00 (base)	
	Improved	566	60.1	1.49 (0.80–2.79)	0.212	1.40 (1.14–1.73)	0.001
Child hand washing score tertiles^§^	Lowest	300	51.0	1.00 (base)		1.00 (base)	
	Middle	394	55.6	1.09 (0.89–1.35)	0.387	0.99 (0.80–1.22)	0.917
	Highest	149	71.8	1.61 (1.26–1.07)	<0.001	1.26 (0.97–1.65)	0.083
Stools disposal	Unsafe	234	50.0	1.00 (base)		1.00 (base)	
	Safe	609	59.4	1.35 (1.10–1.66)	0.005	1.20 (0.96–1.49)	0.105
Topics covered by the HEW counselling	≥ 3	142	67.6	1.00 (base)		1.00 (base)	
	1–2	194	60.3	0.76 (0.78–1.00)	0.052	0.93 (0.70–1.25)	0.646
	0	506	52.6	0.67 (0.53–0.85)	0.001	0.88 (0.68–1.14)	0.348
Vitamin A in past 6 months	No	256	48.0	1.00 (base)		1.00 (base)	
	Yes	587	60.6	1.48 (1.20–1.81)	<0.001	1.05 (0.84–1.30)	0.678

†N in multivariable analysis: 856 with 467 events.

‡N in bivariate analysis

§The proportional hazards assumptions were violated for these variables and the HR therefore should be interpreted as an average HR over the period of follow up.

Abbreviations: CI. Confidence interval, HR, Hazard Ratio; MUAC, mid-upper arm circumference; WHZ, weight-for-height z score; HAZ, height-for-age z-score; HEW, health extension worker

### Summary of key results with cohort subsample using MUAC cut-offs as defined by WHO (2009)

Table 8 shows that overall when the WHO criteria were used, more than a quarter of the cohort deteriorated to SAM (MUAC <11.5cm) and only 51% recovered without experiencing an episode of SAM. Only just over a quarter of those who developed SAM had reached MUAC ≥12.5cm by the end of the follow up.

**Table 8:** Distribution of outcomes based on the WHO (2009) MUAC based operational definitions.

	All children†		SAM Children	
Outcome	n	%	n	%
Recovered and never SAM	394	51.0		
Remained MAM (never SAM)	134	17.3		100.0
Remained MAM during the entire follow up			83	62.0
Recovered during follow up but relapsed to MAM			51	38.0
Total SAM (at least 1 episode)	222	28.7		100.0
SAM at week 28			39	17.6
SAM at least once but recovered			59	26.6
SAM at least once but MAM at week 28			110	49.6
SAM at last visit before death reported			7	3.1
SAM at least once but lost to follow up			7	3.1
Lost to follow up	22	2.9		
Died (without SAM at last visit before death reported)	1	0.1		
Total	773	100.0		

†The 111 children with MUAC <11.5cm at enrolment excluded

Table 9 shows that the incidence of SAM was very high among children of the cohort when the WHO case definition was used with 16 children out of 100 children deteriorating to SAM every month in children who had a MUAC between 11.5 cm and 11.9 cm at enrolment.

**Table 9:** Incidence of Severe Acute Malnutrition when using WHO (2009) MUAC-based case definition of MAM and SAM.

Stratification by MUAC at enrolment	N	No. of episodes	Follow-up time (100 months)†	Incidence rate per 100 person-months‡	95% CI
All	769	218	37.9	5.74	5.03–6.56
12.0–12.4 cm	556	99	30.6	3.23	2.65–3.93
11.5–11.9 cm	213	119	7. 3	16.28	13.60–19.49

† Time from enrolment to first episode of SAM, expressed in units of 100 months.

‡ Incidence of first episode of SAM, expressed per 100 person-months.

Abbreviations: MUAC, mid-upper arm circumference; CI, confidence interval.

Table 10 below shows that in addition to MUAC, weight-for-height/length, child feeding practices, household food insecurity and receiving routine Vitamin A supplementation predicted deterioration to SAM (MUAC <11.5cm). The extended Cox proportional hazards model is presented in Table C of S1 File.

**Table 10:** Predictors of occurrence of severe acute malnutrition (using WHO MUAC cut-off) based on parameters at enrolment: bivariate and Cox proportional hazards analyses ^†^.

Variable	Category	n‡	% SAM	Unadjusted HR (95% C.I.)	P-value	Adjusted HR (95% C.I.)	P-value
Gender	Male	314	28.3	1.00 (base)		1.00 (base)	
	Female	456	29.2	1.04(0.79–1.37)	0.760	1.08(0.82–1.44)	0.571
Age group	≥ 24 months	332	26.8	1.00 (base)		1.00 (base)	
	< 24 months	471	30.2	1.15(0.88–1.51)	0.314	1.26(0.93–1.70)	0.143
Food insecurity	None	253	22.5	1.00 (base)		1.00 (base)	
	Moderate	266	28.2	1.26(0.89–1.78)	0.187	1.09(0.77–1.55)	0.634
	Severe	254	35.4	1.64(1.17–2.30)	0.004	1.39(0.99–1.96)	0.059
Child feeding practices score tertiles	Lowest	148	25.0	1.00 (base)		1.00 (base)	
	Middle	337	31.2	1.34(0.92–1.96)	0.131	1.51(1.02–2.22)	0.039
	Highest	288	27.8	1.16(0.78–1.72)	0.468	1.40(0.93–2.10)	0.105
MUAC category	12.0–12.4 cm	558	18.1	1.00 (base)		1.00 (base)	
	11.5–11.9 cm	215	56.3	4.27(3.27–5.58)	<0.001	3.74(2.82–4.97)	<0.001
WHZ category (Z-score)	≥-2	501	23.3	1.0 (base)		1.00 (base)	
	≥-3 and <-2	176	38.6	1.81(1.34–2.45)	<0.001	1.71 (1.25–2.33)	0.001
	<-3	84	39.3	1.91(1.29–2.83)	0.001	1.56 (1.03–2.36)	0.036
HAZ category (Z-score)	≥-2	259	24.7	1.00 (base)		1.00 (base)	
	≥-3 and <-2	153	28.8	1.23(0.84–1.83)	0.289	1.19 (0.79–1.79)	0.397
	<-3	357	31.6	1.43(1.05–1.95)	0.025	1.34 (0.94–1.31)	0.102
Topics covered by the HEW counselling	≥ 3	132	22.7	1.00 (base)		1.00 (base)	
	1–2	179	31.3	1.48(0.88–1.98)	0.172	1.39(0.88–2.20)	0.156
	0	461	29.5	1.48(0.94–2.31)	0.088	1.14(0.75–1.72)	0.541
Vitamin A in past 6 months	No	223	37.7	1.00 (base)		1.00 (base)	
	Yes	570	25.1	0.60 (0.46–0.79)	<0.001	0.76 (0.57–1.01)	0.062

†N in multivariable analysis: 757 with 215 events.

‡N in bivariate analysis

Abbreviations: CI. Confidence interval, HR, Hazard Ratio; MUAC, mid-upper arm circumference; WHZ, weight-for-height z score; HAZ, height-for-age z-score; HEW, health extension worker

## Discussion

This study has highlighted that nearly half of children who are suffering from MAM at the beginning of the post-harvest season either develop SAM or cross the four months of best food security without recovering. Many are therefore in danger of entering the next hunger season in a highly vulnerable condition. These risks are accentuated amongst those with low MUAC at enrolment. Furthermore, those children who do manage to recover take a long time to do so (9 weeks), and such a prolonged exposure to MAM risks serious negative consequences for their health and development [2]. Our study did not include the most food insecure months and we can assume that the results would be even more alarming if the period had partially or fully covered the hunger season.

### Deterioration to SAM

Preventing deterioration from MAM to SAM is one of the main reasons for setting up SFPs during food crises. 9.3% of our cohort experienced at least one episode of SAM during the follow up, and every month 7.8% of the children enrolled with MUAC between 11.0–11.4 cm deteriorated to SAM. This demonstrates that MAM is a serious public health problem in settings of Ethiopia with no access to SFPs. Two papers have reported deterioration rates from MAM to SAM in SFPs implemented in Ethiopia during hunger seasons in settings classified as food insecure [31,46]. The first study, which evaluates SFP performance in four food insecure regions of Ethiopia, indicates approximately 10% deteriorated from MAM to SAM over 6 months of follow up [31]. This shows that our cohort had the same rate of deterioration to SAM as those children covered by a SFP in much worse food security settings. The second study tested the effectiveness of different specialised foods, reporting a cumulative proportion of 1–2% of MAM children progressing to SAM over 16 weeks despite treatment either with corn-soy blend or RUSF [46]. This highlights the potential to reduce rates of deterioration from MAM to SAM with a well–implemented SFP.

### Comparison with SPHERE standards

SPHERE minimum standards are international benchmarks for programme outcomes in humanitarian emergencies [47]. They provide a useful comparison for our cohort outcomes since if the standards are not met this might indicate additional interventions are needed. Given there was no SFP we were interested to compare whether community and clinic-based health care given to a child in the absence of a SFP would perform as well as the standard care provided to a child in a SFP. SPHERE guidelines for the management of MAM in SFPs set 75% as the minimum standard for recovery [47]. With our observed recovery rate of 54.2% this minimum standard was not met for our cohort, lending support for advocacy of a nutrition specific intervention to improve recovery rates. However, good recovery rates have also proven elusive for many SFPs addressing MAM [48]. For example, the results of an outcome evaluation recently conducted for the World Food Programme (WFP) in Ethiopia found a recovery rate of 49% over 6 months of follow up, even when some children received a food supplement at a dose of 1378 kcal per person per day [31].

SPHERE standards have set <3% as a minimum standard for mortality for children managed with MAM [47]. Although one might expect a MAM cohort not enrolled in a SFP to exceed that minimum standard, our cohort met that criterion (mortality was 0.9%). With our very low level of loss to follow up we have confidence in this estimation. Our observed mortality rate was three-fold lower than that documented in the evaluation of SFPs in four food insecure regions of Ethiopia (2.9%) [31]. In addition, our observed incidence of death equals the widely quoted average under-5 mortality rate for Sub-Saharan countries of 0.44 deaths per 10,000 days [47]. Mason *et al*. compiled under-5 mortality rates in Ethiopia from 76 mortality cross-sectional surveys conducted between 2007–2009 and found an average rate of 0.56 deaths per 10,000 child days [49]. Our cohort may therefore have experienced better survival than that observed in the general population. A possible explanation for the low observed mortality rate could be that our weekly follow up led to rapid referral of children with SAM and other morbidities for treatment, and may point towards the effectiveness of the available health service. Despite the observed low mortality, of concern is the fact that 7 of the 8 deaths occurred in children who had developed SAM at one point during follow up, with four children dying during an episode of SAM. Although it is difficult to make conclusions based on such a limited number of deaths, the figure again highlights the possible high contribution of acute malnutrition to under-5 mortality in Ethiopia and the urgency of preventing the progression of MAM to SAM.

### Predictors of outcomes

Wealth was associated with the evolution of MAM in our study with favourable outcomes observed in children of wealthier households. This has been observed by others in Ethiopia and elsewhere [50,51]. Similarly most of the other predictors of outcomes identified in this study including water drinking source, sanitary disposal of stools, mother’s work index, nutrition counselling and routine vitamin A intake, have been observed before [50–53]. We did not anticipate the inverse relationship of children with mothers reporting the heaviest workload having a better chance of recovery from MAM. It is well documented that increased women’s workload in traditional rural Sub-Saharan society negatively affects their child’s health condition and that if time is made available better child health outcomes can be achieved [54,55]. Our finding suggests the opposite, in that a child’s outcome is negatively affected by a reduction in mothers working time, and that the effect of this reduction is not compensated by the possible increase in time for caring for the child. Indeed, the heavy workload in this context may just be a proxy of better food availability and diversification resulting from the women’s farm production or non-farming income [56]. Anecdotal information collected in Jimma indicated that cash crop revenue that is usually managed by men is rarely used for improving household food security but for investments such as improving household assets and quality of housing, with income from women more likely to be allocated to food. It is also of note that others have found a similar paradoxical relationship between maternal labour saving and children nutrition status in Ethiopia. A retrospective study carried out in rural Ethiopia suggested that labour saving technology is associated with increased fertility in women, being further associated with an increased malnutrition rate [57].

MUAC at enrolment had the strongest association with both recovery from MAM and deterioration to SAM. The data suggests that even during the early weeks of the post-harvest season these children face unacceptable risks for a prolonged period of time, with the lower the enrolment MUAC the higher the risks.

### Stunting and wasting

Two thirds of the children were stunted, with almost half of all children being severely stunted. This suggests a chronically inadequate intake of nutrients (especially that of nutrients related to linear growth) by both pregnant women and young children [1,58]. Compared with an overall stunting prevalence of 41% for Oromia region observed during 2011 [22], the prevalence of stunting was much higher among children of our cohort. This higher prevalence of stunting at enrolment suggests that stunted children are disproportionally more affected by MAM than non-stunted children. This is not surprising because it is well known that stunted children have reduced muscle mass and lower capacity to resist nutritional and infectious shocks [59]. However, it may also reflect the observation documented elsewhere indicating that repeated episodes of acute malnutrition in early childhood decreases linear growth [59,60]. It is also possible that the high prevalence of stunting in our sample could be due to the preferential selection of stunted children when defining MAM based on MUAC, as seen in recent surveys [61].

### Results using subsample meeting WHO (2009) MUAC cut-offs

When reanalysing the results using the WHO (2009) MUAC cut-offs for SAM and MAM the deterioration to SAM almost tripled as the SAM cut-off is now increased to 11.5cm. We still find the same pattern whereby those enrolled with the lowest MUAC have increased risk of deteriorating, and this remains evident even after excluding those with the lowest MUAC category (11.0–11.4cm) from the analysis. These findings help to accentuate the urgency of programmatic reform when comparing results to the international criteria more commonly used outside Ethiopia.

### Programmatic recommendations

It may well not be feasible to have a national intervention targeting all children with MAM using the more commonly utilised cut-off of <12.5cm. However, at the minimum, our findings suggest that an intervention should be considered for those with MUAC <11.5 cm. The change of cut-off for admission into therapeutic feeding to MUAC <11.5 cm may be the most appropriate approach given the high likelihood of children in this group deteriorating. At the global level this higher admission cut-off has already been endorsed by WHO, UNICEF and key government partners in CMAM protocols [28]. Several findings support the notion that children with MUAC <11.5cm are suffering from SAM and require management with OTP protocols. Firstly community-based studies show these children have an increased risk of dying [62]. Secondly, there is evidence that CMAM protocols improve long-term survival of recovered SAM children compared to other children of the same community [63]. The effective scale up and good geographical coverage of SAM treatment in Ethiopia, including in woredas classified as food secure, would support the operational feasibility of this change in admission criteria. Indeed, data from a pilot study in Bangladesh, having a higher caseload of SAM than Ethiopia, showed that shifting to the use of MUAC <11.5cm admission criteria for SAM treatment did not result in an unmanageable increase of workload for community health workers, and the quality of the other services provided remained unaffected [64,65].

Given that children with an enrolment MUAC between 11.5–11.9cm demonstrated a high likelihood of deteriorating or not recovering, there is a clear need to re-assess the current strategy and identify a better approach that could reduce MAM duration and minimise the long-term negative consequences. Given the challenges of countrywide scaling up of SFPs during the critical period covering the hunger period and 12 weeks after harvesting, it might be much easier to admit these children in OTPs because these are already scaled up countrywide with very good coverage. A study in Niger and in Malawi showed that using RUTF for treating MAM improved recovery whilst reducing treatment duration, even when the dose was half that used for treating SAM [66,67]. Also, in a recently published study conducted in Sierra Leone an integrated programme using the same protocol and specialised food product for the treatment of both MAM and SAM was deemed simpler to implement, achieved the same high recovery rate and a higher coverage compared to using two different food products and running SFPs (for MAM) and OTPs (for SAM) separately [8]. However, many different products exist for the treatment of MAM and should be considered in the local context. For example, in Malawi a fortified corn-soy blend with added oil and dry skim milk performed as effectively as two ready-to-use supplementary foods [68].

Another suggested change to CMAM protocol is the adoption of a MUAC discharge criterion for children admitted with MUAC only. At the time of the development of the current Ethiopian national protocols for the management of SAM, there was no data on the use of MUAC for monitoring response to therapeutic feeding. A series of meetings were organised by the Nutrition Advisory Group of the WHO in Geneva from 2010 and 2012. They examined programmatic evidence emerging after 2005 and concluded that MUAC could be used for monitoring response and graduating from treatment, recommending that the use of percentage weight gain be dropped and replaced by MUAC ≥12.5cm for discharge [69–72]. Findings of the present study suggest this policy would minimise the risk of relapses and negative outcomes amongst children who are currently being discharged from OTPs too early.

Feasibility, effectiveness and cost-effectiveness of alternative approaches should also be evaluated, especially those listed in the National Nutrition Programme (NNP) that acknowledge the integration needed between prevention and treatment programmes. Programming tackling childhood stunting and micronutrient deficiencies, particularly focussed from preconception to age two, will also have an impact on prevention of wasting [59]. Such strategies may include child-centred specific nutrition counselling, cash transfers, linkage to social protection services (safety net programme, income generation schemes), a food multimix approach using locally available food, and using specialised food supplements such as fortified blended flours or ready-to-use supplementary foods [12,15,17,18]. Given limited resources targeting individuals and households at risk would likely be required, for example based on food security status, wealth classification or child age.

### Strengths and Limitations

A key strength of our study is its longitudinal design. The prospective weekly home follow up allowed precise estimation of the incidence of acute malnutrition and minimised loss to follow up. However, our findings need to be interpreted in the context of some limitations. Due to our desire to follow children who met the definition of MAM using Ethiopian cut-offs, children were enrolled into the study with MUAC 11.0–11.4cm. Whilst this was an important category of children to obtain data on, for many other countries using the international protocols following WHO (2009) guidelines the children in this lowest MUAC category would already be defined as having SAM.

We envisaged enrolling children who had become low MUAC during a period of acute malnutrition, and not infants born with low birth weight who had not recently deviated from their growth pattern. The proportion of low birth weight due to premature delivery or intrauterine growth retardation is known to be around 11% of infants for the Jimma zone [73]. We could not get reliable data on birth weight and past growth pattern, potentially categorising some children as having MAM when they may have been low birth weight children not experiencing a recent weight loss episode. We suspect that the proportion of such children would not have significantly altered our conclusions. Resource constraints precluded a longer duration of follow up and a control group of well-nourished children. It may have been more appropriate to follow the cohort over a full year to explore how children who did not recover during the period of best food security fared when entering the next hunger season. A control would have helped us quantify the incidence of new MAM cases over the time frame, and made a stronger comparison for deterioration to SAM results. Future research addressing the same question could start with a cohort of children, including both well-nourished and those with MAM, recruited at the end of the best food security season and followed up for a year. We may have underestimated the level of adverse outcomes, including mortality, by covering only the period with reduced nutritional risks, and therefore our results should be interpreted as the ‘best case’ scenario. Finally, we cannot generalise our results to all woredas having no access to SFPs in Ethiopia. For ethical reasons we chose woredas with functioning health services, including CMAM programmes for SAM treatment. Again, we feel our results represent the best scenario and woredas without such good coverage of health care may experience worse outcomes.

## Conclusion

Our results indicate that children with MAM during the post-harvest season in an area with no access to SFPs experience an unacceptably high incidence of SAM and a low recovery rate. This highlights the need to re-assess the current strategy for addressing MAM in these areas. The results suggest that the current approach of not having a targeted nutrition specific intervention to address MAM in this context place children with MAM at excessive risk of adverse outcomes. As the study was implemented during the best period for food security, we were not expecting such a high rate of deterioration and assumed that there would be sufficient food in households to ensure recovery from MAM, yet for close to half of these children this recovery did not happen. It is likely that if the study had been implemented during the hunger period, the rate of deterioration to SAM would have been even higher, with a concomitant increase in mortality. It is concerning to learn that such a high proportion of children with MAM will enter the next hunger season in such a vulnerable condition, with likely consequences of increased risk of SAM and death. Additional preventive and curative approaches should urgently be considered, particularly aimed at children with low MUAC, taking into account feasibility within the resource constraints of middle and low-income public health programming.

## Supporting Information

S1 File Extended Cox proportional hazards models for the predictors of outcomes. (PDF)

S1 Table The Infant and Child Feeding Index Scoring Table. (PDF)
