# Supplementary material for: Children with Moderate Acute Malnutrition with No Access to Supplementary Feeding Programmes Experience High Rates of Deterioration and No Improvement: Results from a Prospective Cohort Study in Rural Ethiopia
Source: PLoS One. 2016 Apr 21;11(4):e0153530. doi: 10.1371/journal.pone.0153530 (PMC4839581; doi:10.1371/journal.pone.0153530)
Supplement: S1 File — (PDF) [file pone.0153530.s001.pdf]

Supplemental Table A: Predictors of occurrence of severe acute malnutrition based on parameters at enrolment: Standard and extended Cox proportional hazards analyses†

(Total n=869 with 80 events)

**Variable**	**Category**	**Standard Cox-PH model HR (95% C.I.)**	**P-value**	**Extended Cox-PH model HR (95% C.I.)**	**P-value**
**Main effect**					
Gender	Male	1.00 (base)		1.00 (base)	
	Female	0.98 (0.61-1.57)	0.937	0.53 (0.24-1.15)	0.107
Age group	≥ 24 months	1.00 (base)		1.00 (base)	
	< 24 months	1.32 (0.79-2.20)	0.292	1.34 (0.80-2.24)	0.269
Food insecurity	None	1.00 (base)		1.00 (base)	
	Moderate	1.33 (0.75-2.36)	0.335	1.31 (0.74-2.33)	0.353
	Severe	1.37 (0.78-2.39)	0.273	1.35 (0.77-2.36)	0.291
Child feeding practices score tertiles	Lowest	1.00 (base)		1.00 (base)	
	Middle	1.33 (0.71-2.52)	0.374	1.33 (0.70-2.51)	0.380
	Highest	1.58 (0.83-3.01)	0.161	1.57 (0.82-2.99)	0.170
MUAC category§	12.0 – 12.4 cm	1.00 (base)		1.00 (base)	
	11.5 – 11.9 cm	3.60 (1.91-6.80)	<0.001	3.60 (1.91-6.79)	<0.001
	11.0 - 11.4 cm	12.31(6.63-22.85)	<0.001	36.42(13.57-97.73)	<0.001
WHZ category§ (Z-score)	≥-2	1.00 (base)		1.00 (base)	
	≥-3 and <-2	1.35 (0.80-2.38)	0.308	1.33 (0.79-2.25)	0.280
	<-3	1.37 (0.74-2.53)	0.255	1.37 (0.74-2.53)	0.318
HAZ category (Z-score)	≥-2	1.00 (base)		1.00 (base)	
	≥-3 and <-2	0.88 (0.42-1.84)	0.738	0.87 (0.42-1.82)	0.718
	<-3	1.38 (0.78-2.44)	0.270	0.80 (0.35-1.80)	0.586
Topics covered by the HEW counselling	≥ 3	1.00 (base)		1.00 (base)	
	1-2	0.89 (0.38-2.10)	0.789	0.88 (0.37-2.08)	0.777
	0	1.63 (0.80-3.32)	0.181	1.63 (0.80-3.33)	0.182
Vitamin A in past 6 months	No	1.00 (base)		1.00 (base)	
	Yes	1.10 (0.69-1.76)	0.690	1.11 (0.69-1.78)	0.666
**INTERACTIONS**					
Female*time				1.10 (0.99-1.21)	0.063
HAZ<-3*time				1.08 (0.99-1.19)	0.088
MUAC (cm) 11.0 - 11.4*time				0.84(0.74-0.96)	0.011

†Standard Cox proportional analysis assumes proportional hazard assumption for all explanatory variables while extended cox proportional hazard analysis take into account the non-proportionality of MUAC category gender and HAZ category.

Abbreviations: SAM, Severe Acute Malnutrition; CI, Confidence interval; HR, Hazard Ratio; MUAC, mid-upper arm circumference; WHZ, weight-for-height z score; HAZ, height-for-age z-score; HEW, health extension worker

Supplemental Table B: Predictors of recovery based on parameters at enrolment: Standard and extended Cox proportional hazards analyses†

(Total n=856 with 467 events)

**Variable**	**Category**	**Standard Cox-PH model HR (95% C.I.)**	**P-value**	**Extended Cox-PH model HR (95% C.I.)**	**P-value**
**Main effect**					
Gender	Male	1.00 (base)		1.00 (base)	
	Female	0.84 (0.70-1.02)	0.083	0.85 (0.70-1.03)	0.097
Age group	≥ 24 months	1.00 (base)		1.00 (base)	
	< 24 months	0.78 (0.64-0.96)	0.020	0.80 (0.65-0.99)	0.043
Food insecurity	None	1.00 (base)		1.00 (base)	
	Moderate	0.87 (0.70-1.09)	0.228	0.89 (0.71-1.11)	0.307
	Severe	0.81 (0.63-1.03)	0.085	0.83 (0.66-1.06)	0.143
Mother's working index	Least heavy	1.00 (base)		1.00 (base)	
	Middle	0.97 (0.76-1.24)	0.825	0.96 (0.75-1.23)	0.735
	Most heavy	1.75 (1.40-2.18)	<0.001	1.78 (1.43-2.23)	<0.001
MUAC category	12.0 – 12.4 cm	1.00 (base)		1.00 (base)	
	11.5 – 11.9 cm	0.40 (0.31-0.52)	<0.001	0.24 (0.14-0.39)	<0.001
	11.0 - 11.4 cm	0.24 (0.16-0.36)	<0.001	0.08 (0.03-0.21)	<0.001
WHZ category (Z-score)	≥-2	1.00 (base)		1.00 (base)	
	≥-3 and <-2	0.70 (0.56-0.88)	0.003	0.52 (0.34-0.78)	0.002
	<-3	0.69 (0.49-0.98)	0.036	0.71 (0.50-1.00)	0.054
HAZ category (Z-score)	≥-2	1.00 (base)		1.00 (base)	
	≥-3 and <-2	1.14 (0.89-1.48)	0.298	1.15 (0.89-1.49)	0.284
	<-3	0.89 (0.71-1.13)	0.351	0.90 (0.71-1.14)	0.405
Topics covered by the HEW counselling	≥ 3	1.00 (base)		1.00 (base)	
	1-2	0.93 (0.70-1.25)	0.646	0.93 (0.70-1.25)	0.656
	0	0.88 (0.68-1.14)	0.348	0.88 (0.68-1.14)	0.349
Drinking water source	Unimproved	1.00 (base)		1.00 (base)	
	Improved	1.40 (1.14-1.73)	0.001	1.37 (1.12-1.69)	0.003
Wealth quintile	1-4	1.00 (base)		1.00 (base)	
	5 (Richest)	1.34 (1.07-1.68)	0.010	1.33 (1.06-1.67)	0.012
Stools disposal	Unsafe	1.00 (base)		1.00 (base)	
	Safe	1.20 (0.96-1.49)	0.105	1.18 (0.95-1.47)	0.134
Child hand washing score tertiles	Lowest	1.00 (base)		1.00 (base)	
	Middle	0.99 (0.80-1.22)	0.917	0.78 (0.56-1.11)	0.168

Variable	Category	Standard Cox-PH model HR (95% C.I.)	P-value	Extended Cox-PH model HR (95% C.I.)	P-value
	Highest	1.26 (0.97-1.65)	0.083	1.21 (0.91-1.59)	0.187
Vitamin A in past 6 months	No	1.00 (base)		1.00 (base)	
	Yes	1.05 (0.84-1.30)	0.678	1.03 (0.83-1.27)	0.816
**INTERACTIONS**					
MUAC category (cm)	11.5-11.9*time			1.10 (0.99-1.21)	0.063
	11.0 - 11.4*time			1.10 (0.99-1.21)	0.063
Child hand washing score tertiles	Middle*time			1.02 (0.99-1.05)	0.108
WHZ category (Z-score)	≥-3 and <-2*time			1.03 (1.00-1.06)	0.063

†Standard Cox proportional analysis assumes proportional hazard assumption for all explanatory variables while extended cox proportional hazard analysis take into account the non-proportionality of MUAC category child hand washing score and WHZ category analysis

Abbreviations: SAM, Severe Acute Malnutrition; CI, Confidence interval; HR, Hazard Ratio; MUAC, mid-upper arm circumference; WHZ, weight-for-height z score; HAZ, height-for-age z-score; HEW, health extension worker

Supplemental Table C: Predictors of occurrence of severe acute malnutrition based on parameters at enrolment, selecting children fulfilling the WHO (2009) definition of MAM: Standard and extended Cox proportional hazards analyses†

(Total n=757 with 215 events)

Variable	Category	Standard Cox-PH model HR (95% C.I.)	P-value	Extended Cox-PH model HR (95% C.I.)	P-value
**Main effect**					
Gender	Male	1.00 (base)		1.00 (base)	
	Female	1.08(0.82-1.44)	0.571	1.08(0.81-1.43)	0.602
Age group	≥ 24 months	1.00 (base)		1.00 (base)	
	< 24 months	1.26(0.93-1.70)	0.143	1.25 (0.92-1.70)	0.147
Food insecurity	None	1.00 (base)		1.00 (base)	
	Moderate	1.09(0.77-1.55)	0.634	0.76(0.46-1.23)	0.262
	Severe	1.39(0.99-1.96)	0.059	1.39(0.98-1.96)	0.061
Child feeding practices score tertiles	Lowest	1.00 (base)		1.00 (base)	
	Middle	1.51(1.02-2.22)	0.039	1.49(1.01-2.20)	0.043
	Highest	1.40(0.93-2.10)	0.105	1.73(1.03-2.90)	0.037
MUAC category	12.0 – 12.4 cm	1.00 (base)		1.00 (base)	
	11.5 – 11.9 cm	3.74(2.82-4.97)	<0.001	3.78(2.85-5.02)	<0.001
WHZ category (Z-score)	≥-2	1.00 (base)		1.00 (base)	
	≥-3 and <-2	1.71 (1.25-2.33)	0.001	1.70 (1.25-2.32)	0.001
	<-3	1.56 (1.03-2.36)	0.036	1.59 (1.05-2.39)	0.027

Variable	Category	Standard Cox-PH model HR (95% C.I.)	P-value	Extended Cox-PH model HR (95% C.I.)	P-value
HAZ category (Z-score)	≥-2	1.00 (base)		1.00 (base)	
	≥-3 and <-2	1.19 (0.79-1.79)	0.397	1.21 (0.81-1.82)	0.354
	<-3	1.34 (0.94-1.31)	0.102	1.81 (1.13-2.91)	0.013
Topics covered by the HEW counselling	≥ 3	1.00 (base)		1.00 (base)	
	1-2	1.39(0.88-2.20)	0.156	1.39(0.88-2.19)	0.161
	0	1.14(0.75-1.72)	0.541	0.77(0.46-1.30)	0.335
Vitamin A in past 6 months	No	1.00 (base)		1.00 (base)	
	Yes	0.76 (0.57-1.01)	0.062	0.76 (0.57-1.02)	0.069
**INTERACTIONS**					
Food insecurity	Moderate*time			1.06 (1.00-1.12)	0.027
Topics covered by the HEW counselling	None*time			1.07 (1.01-1.13)	0.021
HAZ category (Z-score)	HAZ<-3*time			0.95 (0.90-1.00)	0.050
Child feeding practices score tertiles	Highest*time			0.96(0.91-1.02)	0.194

†standard Cox proportional analysis assumes proportional hazard assumption for all explanatory variables while extended cox proportional hazard analysis take into account the non-proportionality of food insecurity, session of HEW counselling, HAZ category and child feeding practices score tertiles.

Abbreviations: SAM, Severe Acute Malnutrition; CI, Confidence interval; HR, Hazard Ratio; MUAC, mid-upper arm circumference; WHZ, weight-for-height z score; HAZ, height-for-age z-score; HEW, health extension worker
