# Supplementary material for: Children with Moderate Acute Malnutrition with No Access to Supplementary Feeding Programmes Experience High Rates of Deterioration and No Improvement: Results from a Prospective Cohort Study in Rural Ethiopia
Source: PLoS One. 2016 Apr 21;11(4):e0153530. doi: 10.1371/journal.pone.0153530 (PMC4839581; doi:10.1371/journal.pone.0153530)
Supplement: S1 Table — (PDF) [file pone.0153530.s002.pdf]

Infant and Young Child Feeding Index (ICFI) Scoring Table

	Age Group (Months)							
	**6-8**		**9-11**		**12-35**		**36-59**	
	**Value**	**Score**	**Value**	**Score**	**Value**	**Score**	**Value**	**Score**
**Breastfed (24 hours)**	Yes	+2	Yes	+2	Yes	+1	Yes	0
**Food Groups (24 hours)**	1<br>≥2	+1<br>+2	1 or 2<br>≥3	+1<br>+2	2 or 3<br>≥4	+1<br>+2	3 or 4<br>≥5	+2<br>+3
**Meal frequency (24 hours)**	1<br>≥2	+1<br>+2	1 or 2<br>≥3	+1<br>+2	2<br>3<br>≥4	+1<br>+2<br>+3	2<br>3<br>≥4	+1<br>+2<br>+3

For more information see Guevarra E, Siliing K, Chiwile F, Mutunga M, Senesie J, Beckley W, et al. IYCF assessment with small-sample surveys - A proposal for a simplified and structured approach. Field Exchange. 2014;47: 60–70.
